# Is the Impact Factor Driving the Demise of Symposium-Based Books?

**DOI:** 10.1093/jisesa/iez093

**Published:** 2019-10-06

**Authors:** Phyllis G Weintraub

**Affiliations:** Agricultural Research Organization, Gilat Research Center, D. N. Negev, Israel

Special collections or special journal issues and books resulting from symposia in scientific meetings have an important function in assembling relevant information on a specific topic. The *Journal of Insect Science* has published a special collection every year for the last 3 years.

**Fig. 1.**
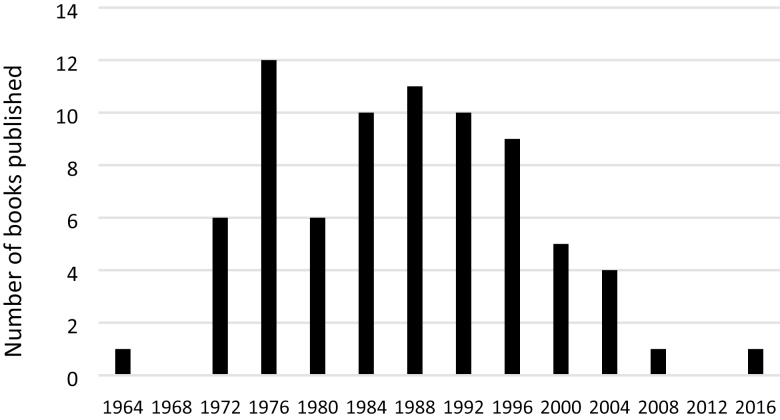
Internet results of books published from 1910 to 2012 (‘International Congress of Entomology’ AND book AND symposium); 2016 datum is a result of symposium organizer survey. No books were found for 1968 and 2012 Congresses.

After joining the team working on a book about the International Congress of Entomology (ICE), which held its last meeting in conjunction with the Entomological Society of America and other international entomological organizations in 2016, I noticed there was a long and extensive publication record of books based on symposia at ICE meetings until recently. A look at the trends from ICE meetings (Fig. 1) reveals that publications of symposium-based books have been declining rapidly since 2000. The first book I could find was published after the 1964 Congress in Washington, DC, and the number of published books steadily grew in the 1970s and 1980s but experienced a decline at the turn of the century. This observation caused me to ponder the causes for the dramatic change; after all, in the electronics age, publishing is much, much easier than 30 years ago when ICE book publishing peaked. I postulate a number of potential causes, though the impact of each may have changed over the past 20 years:

1. Discoverability and accessibility. There is an enormous amount of information available online, and this information can be easily accessed and searched. Publishing in an online journal makes content readily available. A book, by its very nature, cannot be as easily searched, but must be read page by page. Book indexes will generally indicate where to start looking, but electronic searches bring you to exact phrase and words and very quickly. Books are simply not as easily searchable unless online, but searching for e-books online is challenging in itself. Therefore, authors might tend toward publishing in journals so that their content is more discoverable and accessible.
2. Speed of technical and scientific changes. Advances in scientific techniques are moving forward very quickly, which would render books obsolete quickly, as opposed to 25 years ago when changes took place much more slowly. If we examine something like the development of sequencing genomes, initially it took a multitude of researchers laborious weeks to manually sequence, but research and technology have undergone a multitude of refinements in a couple of decades and developed to the point where a DNA sequence can be determined automatically in a matter of hours. This speed of technique development renders a book obsolete after only a short time.
3. Subject breadth. At the heyday of publishing ICE symposia as books, the subject areas of symposia were much broader than they are now; for instance, a symposium on biological control now is often narrowed to a specific group of predators, or parasitoids of a subfamily, or even a limited number of crop plants. The number of symposia in the 1968 meeting was 14; in 2016, there were 298. Possibly the subject areas of symposia today are so specific that publishers might feel the potential audience is too small to warrant publication costs.
4. Social factors. Many/most employers want researchers to show how they are contributing to science on national and international levels. Organizing a symposium is a very visible means of contributing and involves a large number of other researchers both in the planning and executing phases as well as the delivery audience. Writing and editing a book generally takes much longer than putting together a group of journal articles, and most critically a book has no impact factor.

The 2016 ICE/ESA meetings had the largest number of participants and symposia, yet like the 2012 Congress, I could not find any published symposia-based books after extensive internet searches. Therefore, all symposia organizers were contacted electronically and asked if they published a book/e-book on their symposium. The results of these inquires (responses from ca. 25% of all symposium organizers) did reveal one published book and one published e-book. The remainder of the 69 people who answered fell into two groups: the vast majority responded that they had not published, some commenting that they never intended to, or there was not enough time or interest; followed by a much smaller group that indicated that participants published independently in a variety of journals or as a journal special issue (six publications). In written responses and subsequent discussions with colleagues, it became clear that the choice of publishing in journals was driven by ‘the tyranny of impact factors’ (Colquhoun 2003).

The *Journal of Insect Science* accepts manuscripts by focusing on the quality and uniqueness of the science. In 2019 it received its highest impact factor (1.446) and ranked 37th of 98 in entomological journals. Furthermore, we are interested in creating more special collections (see current collections at https://academic.oup.com/jinsectscience/pages/special_collections) of articles around topics connected either to a symposium or as an unaffiliated collection and welcome proposals.
